# Intralymphatic glutamic acid decarboxylase administration in type 1 diabetes patients induced a distinctive early immune response in patients with DR3DQ2 haplotype

**DOI:** 10.3389/fimmu.2023.1112570

**Published:** 2023-02-02

**Authors:** Sara Puente-Marin, Fabrícia Dietrich, Peter Achenbach, Hugo Barcenilla, Johnny Ludvigsson, Rosaura Casas

**Affiliations:** ^1^ Division of Pediatrics, Department of Biomedical and Clinical Sciences, Faculty of Medicine and Health Sciences, Linköping University, Linköping, Sweden; ^2^ Institute of Diabetes Research, Helmholtz Munich, German Research Center for Environmental Health, Munich, Germany; ^3^ Technical University Munich, School of Medicine, Forschergruppe Diabetes at Klinikum rechts der Isar, Munich, Germany; ^4^ Crown Princess Victoria Children´s Hospital, Linköping University, Linköping, Sweden

**Keywords:** immunotherapy, autoantigen, GAD-alum, type 1 diabetes, DR3DQ2 haplotype, intra-lymphatic treatment, lymph node

## Abstract

GAD-alum given into lymph nodes to Type 1 diabetes (T1D) patients participating in a multicenter, randomized, placebo-controlled double-blind study seemed to have a positive effect for patients with DR3DQ2 haplotype, who showed better preservation of C-peptide than the placebo group. Here we compared the immunomodulatory effect of GAD-alum administered into lymph nodes of patients with T1D versus placebo with focus on patients with DR3DQ2 haplotype.

**Methods:**

GAD autoantibodies, GADA subclasses, GAD_65_-induced cytokine secretion (Luminex panel) and proliferation of peripheral mononuclear cells were analyzed in T1D patients (n=109) who received either three intra-lymphatic injections (one month apart) with 4 µg GAD-alum and oral vitamin D supplementation (2000 IE daily for 120 days), or placebo.

**Results:**

Higher GADA, GADA subclasses, GAD_65_-induced proliferation and cytokine secretion was observed in actively treated patients after the second injection of GAD-alum compared to the placebo group. Following the second injection of GAD-alum, actively treated subjects with DR3DQ2 haplotype had higher GAD_65_-induced secretion of several cytokine (IL4, IL5, IL7, IL10, IL13, IFNγ, GM-CSF and MIP1β) and proliferation compared to treated individuals without DR3DQ2. Stratification of samples from GAD-alum treated patients according to C-peptide preservation at 15 months revealed that “good responder” individuals with better preservation of C-peptide secretion, independently of the HLA haplotype, had increased GAD_65_-induced proliferation and IL13 secretion at 3 months, and a 2,5-fold increase of IL5 and IL10 as compared to “poor responders”. The second dose of GAD-alum also induced a more pronounced cytokine secretion in “good responders” with DR3DQ2, compared to few “good responders” without DR3DQ2 haplotype.

**Conclusion:**

Patients with DR3DQ2 haplotype had a distinct early cellular immune response to GAD-alum injections into the lymph node, and predominant GAD_65_-induced IL13 secretion and proliferation that seems to be associated with a better clinical outcome. If confirmed in the ongoing larger randomized double-blind placebo-controlled clinical trial (DIAGNODE-3), including only patients carrying DR3DQ2 haplotype, these results might be used as early surrogate markers for clinical efficacy.

## 1 Introduction

Type 1 diabetes (T1D) is a chronic disorder requiring lifelong treatment. Despite intensive treatment, the disease causes substantial morbidity and mortality. Several clinical intervention trials with different approaches to delay or halt disease progression have shown limited efficacy, sometimes accompanied with adverse events (1–6). As an alternative approach to target and limit the systemic side effects of T1D treatments, autoantigens therapies to induce immunologic tolerance has been considered (7, 8).

Use of 65-kD isoform of glutamic acid decarboxylase (GAD_65_) formulated in alum did show encouraging results in a Phase II trial (9), but did not accomplished the expected efficacy in subsequent studies (10, 11). To improve the treatment, GAD-alum was administrated directly into the lymph nodes of patients with recent-onset T1D in an open-label pilot study (DIAGNODE-1) (12). The patients showed decreased C-peptide depletion and HbA1c, less insulin requirement, as well as immunological changes (12). Comparison of participants in DIAGNODE-1 with previous studies, where GAD-alum was administrated subcutaneously at higher doses, showed a somewhat better clinical course and clear differences in the immunological response (13). After these encouraging results, a larger randomized, double-blind, placebo-controlled phase II trial (DIAGNODE-2) was consequently performed. The treatment did not change disease progression in the entire cohort but appeared to be beneficial in patients with DR3DQ2 haplotype, whose preservation of β-cell function was significantly improved compared to placebo treated patients (14).

Here we describe treatment effect on the immune response of patients who participated in the DIAGNODE-2 trial, comparing the effect in the GAD-alum treated patients in relation to the placebo group, as well as focusing on the immunomodulatory effect of GAD-alum in the patients both according to the presence or absence of the DR3DQ2 haplotype and according to the preservation of beta cell function.

## 2 Materials and methods

### 2.1 Study design

DIAGNODE-2 study was a two-arm, multicenter, randomized, double-blind, placebo-controlled trial (NCT03345004) performed at 18 diabetes clinics in the Czech Republic, the Netherlands, Spain, and Sweden as described before (14). Briefly, 109 patients with less than 6 months-onset diabetes aged between ≥12 and <25 years at screening were randomized at a 1:1 ratio stratified by level of serum GADA and by country, receiving one of the following treatments:

- Three intralymphatic injection with 4 μg GAD-alum (Diamyd Medical, Stockholm, Sweden) on days 30, 60, and 90 and 2,000 IE oral vitamin D daily from day 1 to 120.
- Three intralymphatic injection of placebo on days 30, 60, and 90 and oral placebo for vitamin D daily from day 1 to 120.

Patients received 3 injections (1 month apart) of GAD-alum or Placebo into an inguinal lymph node administrated by using ultrasound needle guide, and in addition vitamin D or placebo if serum level of vitamin D was <100 nmol/L (40 ng/mL) at screening.

All patients were evaluated on day 1 (baseline), 1, 2, 3, 6 and 15 months with clinical examination and blood samples. Mixed Meal Tolerance Test (MMTT) (15) was performed at day 1 (baseline) and at 6 and 15 months to measure C-peptide (mean area under the curve [AUC]). Serum C-peptide quantification was determined by dual-sided chemiluminescence immunoassay using two antibodies (Siemens C-peptide assay no. 03649928) by Synlab Pharma Institute (Munich, Germany).

HLA genotyping for all patient was included as an amendment to the original clinical study protocol ahead of treatment unblinding. Individuals genotyping for HLA DR3DQ2 (defined as DRB1 * 03, DQB1 * 02: 01) haplotype at screening were performed centrally by Synlab Pharma Institute (Munich, Germany), using a sequence-specific oligonucleotides kit (One Lambda) (16).

The study was approved by the relevant regulatory authorities and research ethics boards of the participating sites and countries. All participants and their parents/caregivers gave their consent after oral and written information.

### 2.2 Blood samples

For the immunological analysis, blood and serum were collected at all visits. Samples were drawn during the morning hours, and peripheral blood mononuclear cells (PBMCs) were isolated within 24 hours using Leucosep (Greiner Bio One), according to the manufacturer’s instructions at Linköping University, Sweden.

### 2.3 GAD autoantibodies and IgG subclasses

Serum GAD autoantibodies (GADA) levels were assessed by means of enzyme-linked immunosorbent assay (ELISA) by Synlab Pharma Institute (Munich, Germany).

GADA IgG 1, 2, 3, and 4 subclasses were measured by radio-binding assays using IgG subclass-specific biotin-labelled mouse-anti-human monoclonal antibodies bound on Streptavidin Sepharose High Performance beads (GE Healthcare Life Sciences, Freiburg, Germany) (17). Results were expressed as cpm and converted to arbitrary units (AUs) proportional to the cpm of a local standard serum.

### 2.4 Lymphocyte proliferation assay

Proliferative response was analyzed in PBMCs cultured in triplicates in AIM-V medium with β-mercaptoethanol at 37°C in 5% CO_2_, in the presence of 5 μg/mL rhGAD_65_ (Diamyd Medical, Stockholm), CD3/CD28 beads (∼1 bead: 2 cells; Gibco, Life Technologies AS, Oslo, Norway) or buffer alone (Diamyd Medical, Stockholm, Sweden) (18). After 3 days, cells were pulsed for 18h with 0.2 µCi of [^3^H] thymidine/well (PerkinElmer), and thereafter ^3^H incorporation was recorded using a 2450 MicroBeta 2 Plate Counter (PerkinElmer, Waltham, MA, USA). Proliferation was expressed as stimulation index (SI), calculated as the mean of triplicates in the presence of stimulus, divided by the mean of triplicates in buffer alone.

### 2.5 Cytokine secretion assay

Cytokines were quantified both in serum samples and in PBMCs supernatants. PBMCs were cultured in AIM-V medium with β-mercaptoethanol for 7 days at 37°C in 5% CO_2_ in the presence of 5 μg/mL rhGAD_65,_ CD3/CD28 beads (∼1 bead: 2 cells) or buffer alone. The cytokines IL1β, IL4, IL5, IL6, IL7, IL10, IL13, IL17, tumor necrosis factor (TNFα), interferon (IFNγ), GM-CSF, and MIP-1β were measured in cell supernatants using Bio-Plex Pro Cytokine Panel (Bio-Rad, Hercules, CA, USA) according to the manufacturer’s instructions. Same cytokines were quantified in serum. Data was collected using the Luminex 200 ™ (Luminex xMAP™ Corporation, Austin, TX USA). Induced cytokine secretion by GAD_65_ and CD3/CD28 beads was expressed as pg/ml, and calculated by subtracting the spontaneous secretion from cells cultured in buffer alone.

### 2.6 Statistics and software analysis

Immunological data was presented as median with 95% CI following non-normal distribution, and nonparametric tests were applied. For determining differences between groups, the Mann-Whitney test was used, and Wilcoxon test was applied for differences within groups. A probability level of < 0.05 was considered statistically significant. Calculations were performed using GraphPad Prism 8.0.1 for Windows (GraphPad Software, La Jolla, CA, USA).

Clustering heatmap of immune cytokine secretion data were constructed using ClustVis software (https://biit.cs.ut.ee/clustvis/) (19). Columns with similar annotations were collapsed by taking median inside each group. Rows were centered, and unit variance scaling was applied to rows. Both rows and columns were clustered using Euclidean distance and average linkage.

Fast and stimulated C-peptide values were calculated during the clinical trial (14), and presented as mean, following normal distribution.

## 3 Results

### 3.1 Immune response in GAD-alum treated patients

**Figure 1:**
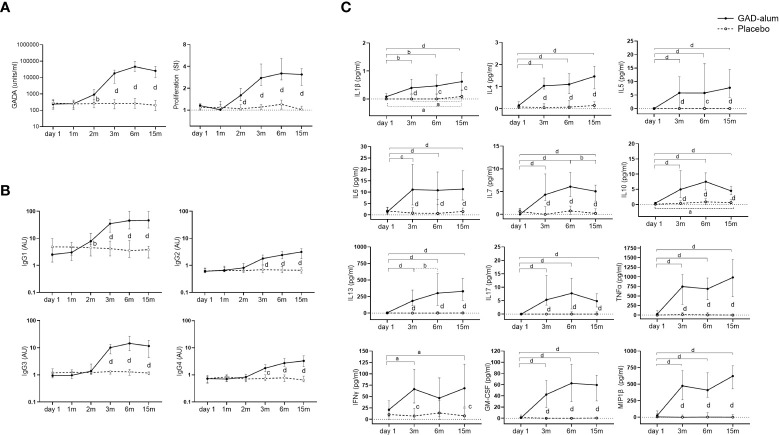
Immune response from day 1 to 15 months in GAD-alum (smooth line, n=56) and Placebo (dashed line, n=52) individuals. **(A)** Median values of GADA (U/ml) and PBMCs proliferative response to GAD_65_ (5 µg/ml). Proliferation is expressed as stimulation index (SI), calculated from the mean of triplicates in the presence of GAD_65_ divided by the mean of triplicates with medium alone. **(B)** Median levels of IgG1, IgG2, IgG3, and IgG4 GADA subclasses, shown as arbitrary units (AUs). **(C)** Cytokine secretion detected by Luminex in PBMCs supernatants after 7-days culture in presence of GAD_65_ (5 µg/ml). Median levels of GAD_65_-induced IL1β, IL4, IL5, IL6, IL7, IL10, IL13, IL17, TNFα, IFNγ, GM-CSF and MIP-1β are given after the subtraction of the spontaneous secretion from each individual, and expressed as pg/ml. Horizontal lines represent the median, and error bars indicate 95% CI. Differences within the same group were calculated using Wilcoxon paired test, and differences between groups were calculated using Mann-Whitney unpaired test. a, b, c, d: p<0.05, p<0.01, p<0.001, p<0.0001.

GADA levels increased in GAD-alum treated patients at 2 months, after the first injection, and remained higher through the study compared to the placebo group (**Figure 1A**). Analysis of GADA IgG subclasses showed that the levels of IgG1 increased at 2 months in actively treated patients, while IgG2, IgG3 and IgG4 were enhanced at 3 months (**Figure 1B**). GADA levels and GADA subclass distribution at baseline (day 1) did not differ between the two groups.

GAD_65_-induced proliferation was also higher in the GAD-alum group following the first injection and remained higher than the placebo group along the study (**Figure 1A**). Levels of GAD_65_-induced IL1β, IL4, IL5, IL6, IL7, IL10, IL13, IL17, TNFα, IFNγ, GM-CSF and MIP1β were enhanced in the PBMC supernatants from the actively treated group following the second GAD-alum dose and remained higher trough the study (**Figure 1C**).

Further stratification of the GAD-alum and placebo groups according to the presence or absence of DR3DQ2 haplotype showed a similar pattern as for the whole cohort. Thus, GADA, GADA IgG1, 2, 3 and 4 subclasses, GAD-induced proliferation and cytokine secretion were higher in GAD-alum treated patients with and without DR3DQ2 compared to placebo individuals with the same haplotype (**Supplementary Figures S1, S2**).

Levels of all analyzed cytokines were similar in PBMC supernatants from placebo and GAD-alum treated subjects, both when cells were cultured in medium alone or in the presence of CD3/CD28. No differences were observed when patients were stratified according DR3DQ2 haplotype. Analysis of cytokines in serum samples did not reveal any difference for any comparison. The same was true for CD3/CD28 induced proliferation (data not shown).

### 3.2 Changes associated to the DR3DQ2 haplotype

**Figure 2:**
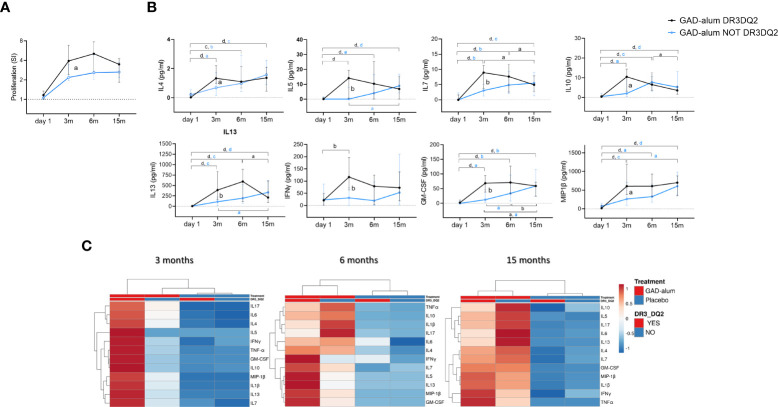
Immune response from day 1 to 15 months in GAD-alum treated patients with (black, n=29) and without (light blue, n=27) DR3DQ2 haplotype. **(A)** Median values of PBMC proliferative response to GAD_65_ (5 µg/ml). Proliferation is expressed as stimulation index (SI), calculated from the mean of triplicates in the presence of GAD_65_ divided by the mean of triplicates with medium alone. **(B)** Cytokine secretion detected by Luminex in PBMCs supernatants after 7-days culture in presence of GAD_65_ (5 µg/ml). Median levels of IL4, IL5, IL7, IL10, IL13, IFNγ, GM-CSF and MIP-1β are given after subtraction of spontaneous secretion from each individual and expressed as pg/ml. Error bars indicate 95% CI. Differences within the same group were calculated using Wilcoxon paired test and differences between groups were calculated using Mann-Whitney unpaired test. a, b, c, d: p<0.05, p<0.01, p<0.001, p<0.0001. **(C)** Heatmap of cytokine secretion induced by GAD_65_ (5 µg/ml) upon *in vitro* PMBCs stimulation of GAD-alum and Placebo treated patients. Columns with similar annotations (Treatment and DR3DQ2 haplotype) were collapsed by taking median inside each group. Both rows and columns are clustered using Euclidean distance and average linkage. Heatmap was performed using Clustvis software. Heatmap data matrix visualizes the values in the cells using a color gradient which gives an overview of the largest and smallest values in the matrix.

Comparison of the immune response in individuals who received GAD-alum stratified according to the DR3DQ2 haplotype showed that GAD_65_-induced proliferation was higher at 3 months in actively treated individuals with DR3DQ2 haplotype compared to those without DR3DQ2 and remained higher along the study (**Figure 2A**). Further, higher levels of GAD_65_-induced IL4, IL5, IL7, IL10, IL13, IFNγ, GM-CSF and MIP1β were observed at 3 months in subjects with the DR3DQ2 haplotype compared to those without DR3DQ2. Differences between the two groups started to wane at 6 months, and no differences were detected at 15 months (**Figure 2B**). Multivariate analyses of the cytokine expression data matrix, represented as a clustering heatmap, illustrates a different cytokine secretion profile in GAD-alum treated patients with DR3DQ2 haplotype at 3 months (**Figure 2C**). GADA levels (data not shown) and GADA IgG subclasses did not differ between the groups (**Supplementary Figure S2**). No difference in the levels of cytokine secretion was observed between samples from patients with and without DR3DQ2 haplotype cultured in medium alone or in the presence of a_CD3/CD28. Cytokine analysis in serum did not reveal any difference between the groups. Proliferation induced by CD3/CD28 was also similar between patients with and without DR3DQ2 haplotype (data not shown).

### 3.3 Immune response in relation to clinical outcome

**Figure 3:**
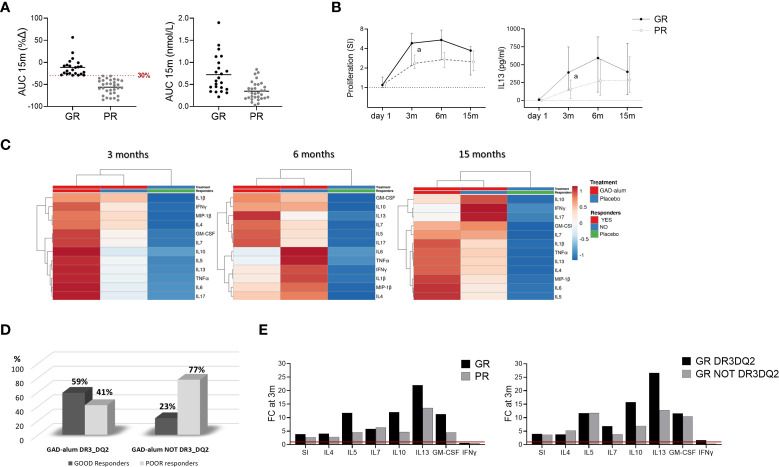
Immune responses in GAD-alum treated patients in relation to Clinical outcome. Patients who received GAD-alum were stratified into Good Responders (GR, n=23, loss < 30% AUC) and Poor Responders (PR, n=32, loss ≥ 30% AUC) according to their C-peptide preservation at 15 months. **(A)** Percentage change of stimulated C-peptide (AUC) from day 1 to 15 months and **s**timulated C-peptide (AUC) nmol/L at 15 months in GR (black) and PR (grey). **(B)** Median values of PBMCs proliferative response and IL13 secretion to GAD_65_ (5 µg/ml) in GR (black) and PR (grey) patients. Proliferation is expressed as stimulation index (SI), calculated from the mean of triplicates in the presence of GAD_65_ divided by the mean of triplicates with medium alone. Median levels of IL13 are given after subtraction of spontaneous secretion from each individual and expressed as pg/ml **(C)** Heatmap of GAD_65-_induced cytokine secretion (5 µg/ml) in GR and PR receiving GAD-alum, and the placebo group. Columns with similar annotations (Treatment and Responders) were collapsed by taking median inside each group. Heatmap was performed using Clustvis software. Heatmap data matrix visualizes the values in the cells using a color gradient which gives an overview of the largest and smallest values in the matrix **(D)** Percentage of GR (black) and PR (grey) individuals in the GAD-alum treated patients with DR3DQ2 (GR: n=17; PR: n=12) or without DR3DQ2 (GR: n=6; PR: n=21). **(E)** Fold change of cytokine secretion and proliferation at 3 months, detected in PBMCs supernatants after 7-days culture in presence of GAD_65_ (5 µg/ml). GAD_65_-induced IL4, IL5, IL7, IL10, IL13, IFNγ and GM-CSF secretion and Proliferation (SI) at 3 months is expressed as fold change from day 1 (red line), between GR (black) and PR (grey) in GAD-alum treated patients and in GR group with (black) and without (grey) DR3DQ2 haplotype. Error bars indicate 95% CI. Differences between groups were calculated using Mann-Whitney unpaired test. a: p<0.05.

To search for immune surrogate markers of clinical efficacy, we looked for the association between remaining C-peptide secretion at 15 months (14) and the immunological markers. For the analysis, GAD-alum treated patients regardless of the DR3DQ2 haplotype, were stratified according to the loss of stimulated C-peptide from day 1 to 15 months, measured as the area under the curve (AUC) as in previous studies (20). “Good Responders” (GR, n=23, AUC loss <30%) and “Poor Responders” (PR, n= 32, AUC loss > 30%) (**Figure 3A**). One patient lacked AUC data at 15 months and was not included in the analysis.

Comparison of the immunological data between GR and PR revealed that GAD_65_-induced proliferation and IL13 secretion were higher at 3 months in GR individuals and remained higher in GR along the study (**Figure 3B**). No other statistically significant differences were observed in univariate analyses, nor any statistically significant association between the immune markers included in the study and stimulated C-peptide (**Supplementary Figure S3**). However, multivariate analysis of the GAD_65_-induced cytokine secretion of GR and PR from the GAD-alum group, and the Placebo group revealed a more pronounced cytokine secretion in GR at 3 months (**Figure 3C**).

To further search for signatures related to the presence or absence of DR3DQ2 haplotype, GR and PR were stratified according to their haplotype (**Figure 3D**). Comparison of the groups did not reveal any statistically significant difference (**Supplementary Figure S3**). However, calculation of the cytokine secretion fold change from day 1 to 3 months revealed more pronounced cytokine secretion, especially IL13 secretion (1.6 times), in GR patients compared to PR in the whole GAD-alum treated group (**Figure 3E**, left). Likewise, the fold change at 3 months in GR with DR3DQ2 haplotype was more pronounced than in GR without DR3DQ2 (**Figure 3E**, right).

## 4 Discussion

The DIAGNODE-2 study constituted the first placebo-controlled trial in which an autoantigen was administrated into the lymph nodes of patients with Type 1 diabetes, and immunological changes observed in GAD-alum treated patients with respect to placebo have not been previously described. Modifications following therapy included an increment of GADA, GADA subclasses, and GAD_65_-induced proliferation and cytokine secretion in GAD-alum treated individuals, with higher titers than the placebo group. GAD_65_-induced secretion of a broad range of cytokines supports the idea of immunomodulation of GAD-specific immune responses following administration of low doses of GAD-alum directly into the lymph nodes.

Although no significant treatment effect was seen in the whole study cohort during the clinical trial, a statistically significant preservation of beta cell function was found in the prespecified subgroup of GAD-alum patients with DR3DQ2 haplotype (14). This is in line with previous findings indicating the influence of HLA genes in T1D (16, 21). Thus, this prompted us to focus on the analysis of the immune responses in GAD-alum treated individuals according to this classification. Strikingly, higher GAD_65_-induced proliferation and secretion of several cytokines was observed in patients with DR3DQ2, compared to those without DR3DQ2 haplotype after the second injection of GAD-alum. Notably IL4, IL5 and IL13, which are major effector cytokines produced by Th2 cells, were among them. It is possible that the generation of an early predominant Th2 and anti-inflammatory response to GAD_65_ might counteract proinflammatory factors and generate an environment where autoreactive Th1 effectors cells could be suppressed, thereby restoring immunological balance. For instances, IL4 mediates signaling to promote Th2 differentiation (22), while IL13 has potent anti-inflammatory activities, both *in vitro* and *in vivo*, and possesses several unique effector functions (23). Together with IL13, IL5 have a central role in immune regulation and differentiation, governing the onset of inflammatory responses and providing signals that help turn off chronic inflammation and protect tissues from ongoing damage (24, 25). Interleukin 10, well known for its potent anti-inflammatory effects, inhibits activation, proliferation, and production of pro-inflammatory cytokines on T cells, in addition to promoting survival, proliferation, differentiation (26). Other cytokines as IFNγ, GM-CSF and IL7 were also part of the earlier cytokine secretion induced by GAD-alum in the DR3DQ2 positive patients. In line with results from our studies, samples from individuals treated with GAD-alum from another clinical trial (10) secreted IL4, IL5 and IL13 Th2-associated cytokines, together with IFN and IL17 (27). The same study also showed that the majority of expanded GAD-specific cells exhibited a hybrid phenotype and were both IL13^+^ and IFN-γ^+^ (27). Thus, it might be argued that responses generated by the treatment represents a non-desirable effect. However, the immune cell network is complex, and cytokines have paradoxical functions. For instances, among the wide-ranging effect of IFNγ on the innate and the adaptive immune system (28), this cytokine can exert different effects depending on their concentrations and microenvironment (29). It has been suggested that low levels of IFNγ may protect animal models from autoimmune diabetes (30, 31). Thus, under the right circumstances, cytokines can exhibit either Th1 or Th2-promoting activities. IL7 is important for B and T cell development and proliferation (32–34), and that has been connected to immune recovery following stem cells transplantation (35). Higher levels of GM-CSF were also detected in the same supernatants, a cytokine that might alter the Th1/Th2 cytokine balance in both directions (36). GM-CSF is known to play an important role in the differentiation of dendritic cells (DCs) and induction of tolerance (37–40) and has a suppressive effect on autoimmune diabetes in mice (41). Indeed, the quality of T cell response induced to vaccination has been connected to multiple cytokines (42), and protective vaccines induced CD4 T cells able to secrete several cytokines while non-protective mostly trigger T cells that produce one or two cytokines (43, 44). Measurement of multiple cytokines simultaneously in cell supernatants by Luminex enabled us monitoring samples from all participants throughout the study, getting a comprehensive depiction of changes induced by intra-lymphatic injections of GAD-alum. In that way, we were able to identify cytokine secretion early after treatment in samples from individuals carrying the DR3DQ2 haplotype with better C-peptide preservation as main finding. However, Luminex does not distinguish the source and extension of cytokines at the single cell level as intracellular cytokine staining does. Thus, as next step we will perform high-dimensional phenotyping in PBMCs selected according to the presence or not of DR3DQ2 haplotype and preservation of C-peptide secretion versus placebo, with focus on the response early after treatment.

The increasing consensus on the heterogenicity of T1D, that can reflect diverse immunological pathways to disease, brings focus to the matter that, as in many other autoimmune diseases, many of the patients participating in T1D clinical trials benefit from the treatments, while others do not (9, 45–47). Most clinical trials evaluating autoantigen immunotherapy have so far failed or shown inconclusive results (9–11, 48, 49). One reason might be that studies often fail to consider T1D as a heterogenous disease (50), and subtypes presenting distinct underlying pathobiological mechanisms, disease endotypes, should be considered in the design and evaluation of clinical trials (51). In that sense, identifying individuals who are most likely to have benefit from a certain treatment will help to develop experimental therapies and personalized approaches. Another big challenge in human studies with autoantigens is optimization of the treatment, including among others the definition of administration routes, doses, frequency and the use or not of adjuvants. The use of additional strategies as DNA-based delivery of autoantigens (52) or the use of tolerogenic dendritic cells pulsed with islet antigen, are also promising strategies as they might boost the efficacy of antigen specific immunotherapy (53, 54).

In an attempt to find biomarkers for clinical response, GAD-alum treated individuals were classified according to their preservation of C-peptide. Comparison of the immunological response revealed that patients displaying a better beta cell preservation had a more pronounced GAD_65_-specific immune response at 3 months. Moreover, this early immune response was more pronounced in individuals with the DR3DQ2 haplotype. Intriguingly, better preservation of residual beta cell function was associated with increased GAD_65_-induced IL13 secretion and proliferation. Thus, it is feasible that early immune responses in individuals with DR3DQ2 haplotype determine the shape of the memory responses to GAD-alum. To generate adequate immunity after vaccination, early innate immune responses are crucial for subsequent signaling for T cell activation and adaptive immune development. Antigen‐specific T cell frequencies peak between days 7 and 14, with only a small percentage of cells surviving as long‐lived memory cells, and their repertoire is determined by selection mechanisms during expansion (55). HLA is an essential checkpoint in antigen presentation and in the shaping of the adaptive immune repertoire (56, 57). The influence of HLA genotype in the immune responses following vaccination has been reported for other vaccines as rubella (58) and anthrax vaccine (59). Although activation of regulatory mechanisms capable of suppressing harmful autoreactive responses is a desired effect of antigen-specific immunotherapies (60), this hypothesis has been generated from experimental animal models but has not yet been demonstrated in humans so far. Deep phenotyping of major immune cell populations in a previous pilot trial showed that the immunomodulatory effect of GAD-alum injections into the lymph nodes did no induced any effect on Tregs, in line with results from previous studies (12, 61–63). Changes induced by the treatment included instead expansion of follicular helper T cells (Tfh), and enhancement of CD8+ T cells with Th2 phenotype in parallel to increased exhausted CD8+T cells (61). Characterization of GAD-specific CD4 T cell clones from 4 patients who received GAD-alum (10) showed that the majority of the clones had characteristics of bifunctional Th1/Th2 cells, leading to the speculation that they might lack immune-modulating properties and hence may not be capable of modifying autoimmunity in a clinically favorable way (27). Thus, it is possible that the therapy does not necessarily induce tolerance or unresponsiveness but rather modulates the quality of the existing immune response to the antigen, resulting in the reduction of deleterious effector functions.

In a previous study we have shown that administration of GAD-alum subcutaneously induced an early Th2-skewed immune response associated to clinical benefit (64). Inconsistent results from following trials led to the search of an alternative approach to improve treatment efficacy, by direct administration of GAD-alum into the lymph nodes (12, 14, 20). The immunological impact of the antigen can be improved by this approach, but it is known that administration route of antigens may not only govern the strength but also the type of the immune response (65). Intra-lymphatic administration delivers more antigen to the site of immune response induction, and difference in antigen dose available for stimulation of antigen-specific T cells may also lead to a predominant Th2 response in responder individuals (55).

Although better preservation of C-peptide and an improvement in several clinical parameters was observed in GAD-alum treated patients with DR3DQ2 haplotype (14), our exploratory analysis showed that some of them has a faster loss of c-peptide secretion. For the analysis, GAD-alum treated individuals were classified as “Good responders” and “Poor responders” according to their preservation of C-peptide (14, 20). However, we should keep in mind that the differences in preservation of beta cell function can sometimes be explained by faster disease progression and baseline characteristics in some individuals, and not directly related to the treatment. It is also likely that pre-existing characteristics other than the HLA genotype might determine the immunological and clinical impact of GAD-alum treatment. Indeed, a possible manifestation of endotypes in T1D was evidenced by birth cohort studies wherein the emergence of autoantibodies towards insulin and GAD_65_ appears to be linked with the HLA DR4DQ8 and DR3DQ2 haplotypes respectively (51, 66). It is feasible that the quality and size of specific immune responses triggered by GAD-alum is determined both by the direct administration into the lymph node and pre-existing phenotypes and genotypes in individuals who benefit from the treatment. For instance, the influence of genetics on the enhancement of effector response to antigens might be related to polymorphisms in the IL-2 and/or TCR signaling pathway (67), rendering T cells more responsive to low levels of stimuli and resulting in the increase of the effective pool size of potentially antigen-responsive precursors. Given the functional role of MHC molecules in the initiation of the immune response, the DR3DQ2 haplotype may also influence the number of GAD_65_ peptides presented by antigen presenting cells. It might also be possible that antigen-specific memory T cells have to compete with preexisting cells for survival factors. Thus, higher frequency of effector T cells when autoantigens are administered might not be desirable, as shown in a in a previous study where individuals with poor response to GAD-alum had a predominant proportion of CD4+ TEM pre-treatment (12). These are important questions that should be addressed in future studies. Our results highlight the importance of incorporating biomarkers into clinical trials. Immune surrogates associated to the response to interventions can be useful in the design of sequential trials and accelerate the development of effective immunotherapies for T1D (60, 68).

In conclusion, here we show that patients with DR3DQ2 haplotype had an early immune response to GAD-alum injections into the lymph node, and predominant GAD_65_-induced IL13 secretion and proliferation that seems to be associated to a better clinical outcome. Better preservation of residual beta cell function was associated with increased GAD_65_-induced IL13 secretion and proliferation. Our results require confirmation in the ongoing larger randomized double-blind placebo-controlled clinical trial (DIAGNODE-3) including only patients carrying DR3DQ2 haplotype. If confirmed, our findings might be used as early surrogate markers for clinical efficacy. It is possible that further immunological or genetic parameters can improve patient selection in future T1D therapies with autoantigens.

## Data availability statement

The raw data supporting the conclusions of this article will be made available by the authors, without undue reservation.

## Ethics statement

The studies involving human participants were reviewed and approved by the relevant regulatory authorities and research ethics boards of the participating sites and countries. Written informed consent to participate in this study was provided by the participants’ legal guardian/next of kin.

## Author contributions

RC designed the experiments. SP-M, FD, and PA performed experiments. SP-M and HB analyzed the data. SP-M and RC interpreted the results and wrote the manuscript. JL conceived DIAGNODE-2 study, recruited, and followed patients. All authors contributed to the article and approved the submitted version.
