# Supplementary material for: Intralymphatic glutamic acid decarboxylase administration in type 1 diabetes patients induced a distinctive early immune response in patients with DR3DQ2 haplotype
Source: Front Immunol. 2023 Feb 2;14:1112570. doi: 10.3389/fimmu.2023.1112570 (PMC9933867; doi:10.3389/fimmu.2023.1112570)
Supplement: Supplementary file 1 [file DataSheet_1.docx]

Supplementary Material

##
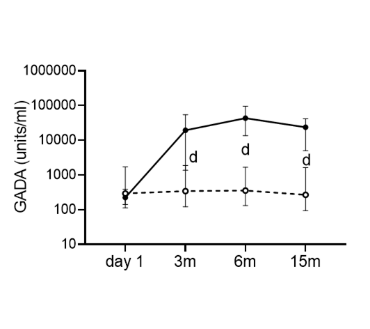

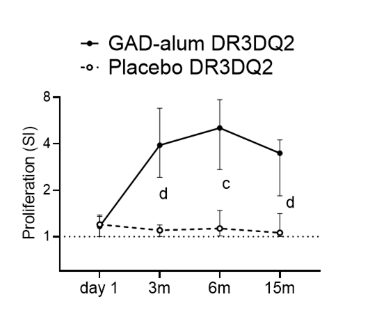

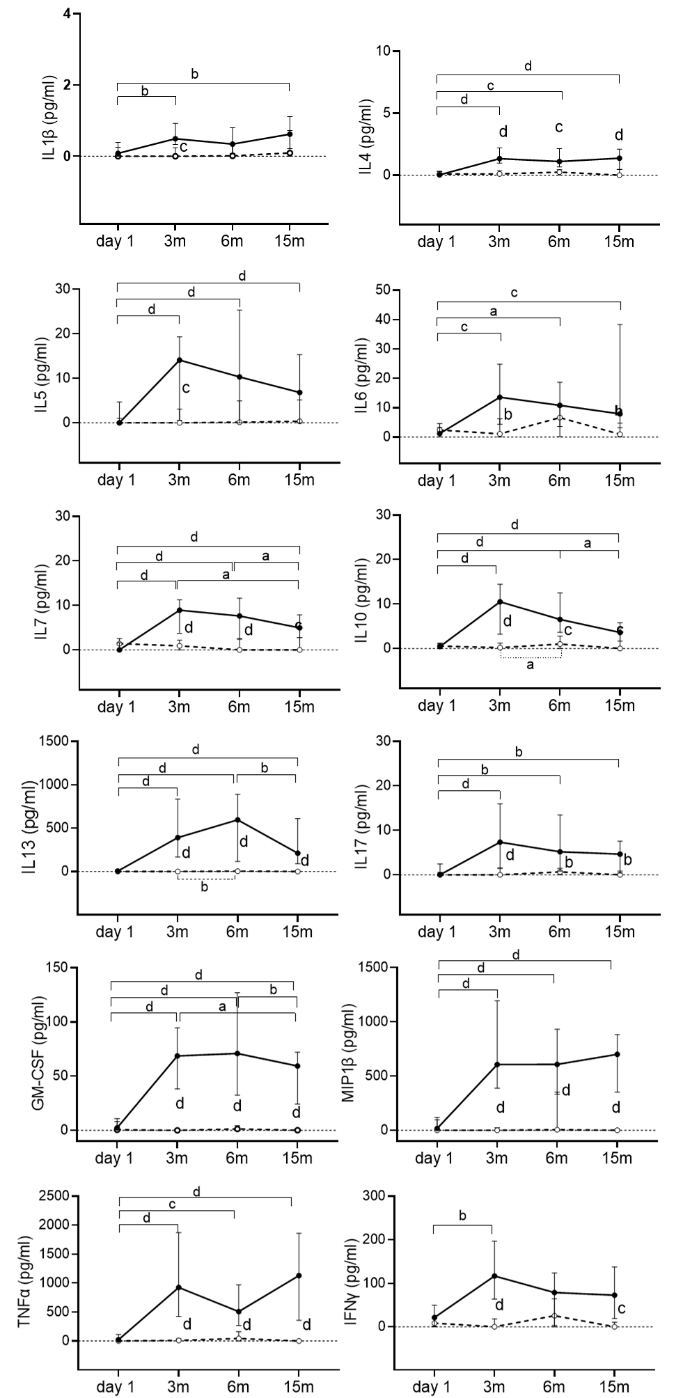

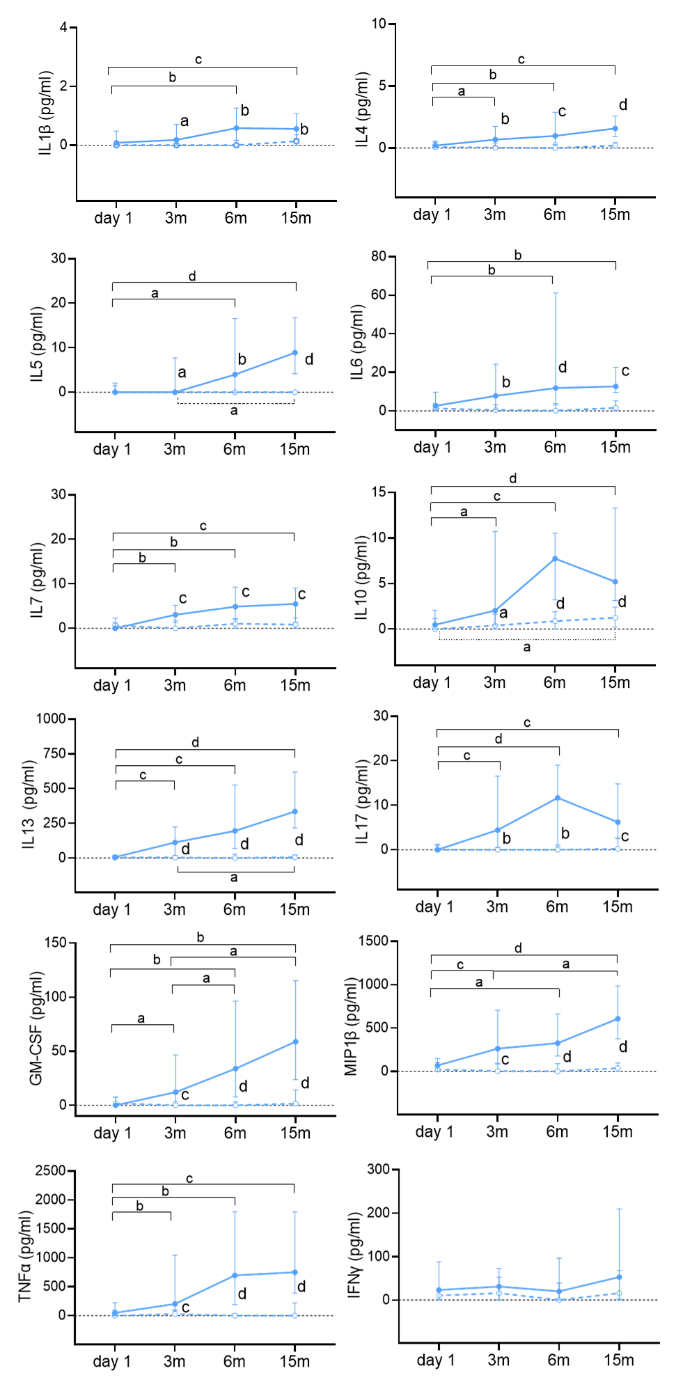

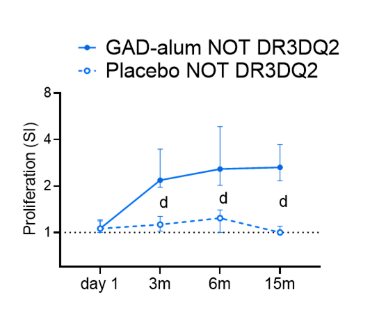

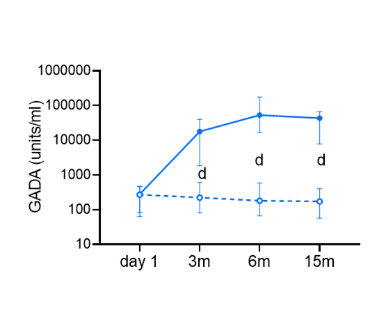
SupplementaryFigures

**A1**

**B2**

**B1**

**B3**

**A3**

**A2**

**A1**

**Supplementary Figure 1:** Immune response from day 1 to 15 months in GAD-alum (n=29) and Placebo (n=19) treated patients with DR3DQ2 (black) and in GAD-alum (n=27) and Placebo (n=32) treated patients without DR3DQ2 (light blue) respectively. **(A1, B1)** Median GADA titers (U/ml). **(A2, B2)** Median values of PBMC proliferative response to GAD_65_ (5 µg/ml). Proliferation is expressed as stimulation index (SI), calculated from the mean of triplicates in the presence of GAD_65_ divided by the mean of triplicates with medium alone. **(A3, B3)** Cytokine secretion detected by Luminex in PBMCs supernatants after 7-days culture in presence of GAD_65_ (5 µg/ml). Median levels of GAD_65_-induced IL1β, IL4, IL5, IL6, IL7, IL10, IL13, IL17, TNFα, IFNγ, GM-CSF and MIP1β are given after the subtraction of the spontaneous secretion from each individual and expressed as pg/ml. Horizontal lines represent the median, and error bars indicate 95% CI. Differences within the same group were calculated using Wilcoxon paired test, and differences between groups were calculated using Mann-Whitney unpaired test. a, b, c, d: p<0.05, p<0.01, p<0.001, p<0.0001.

**A**

**
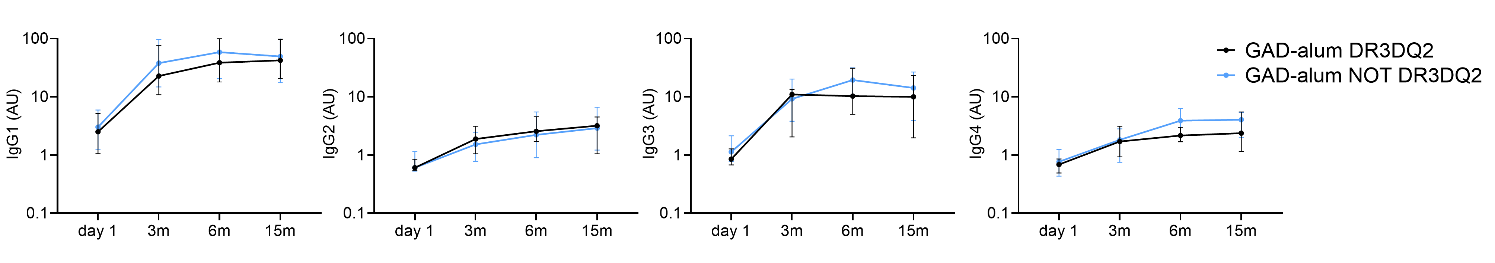

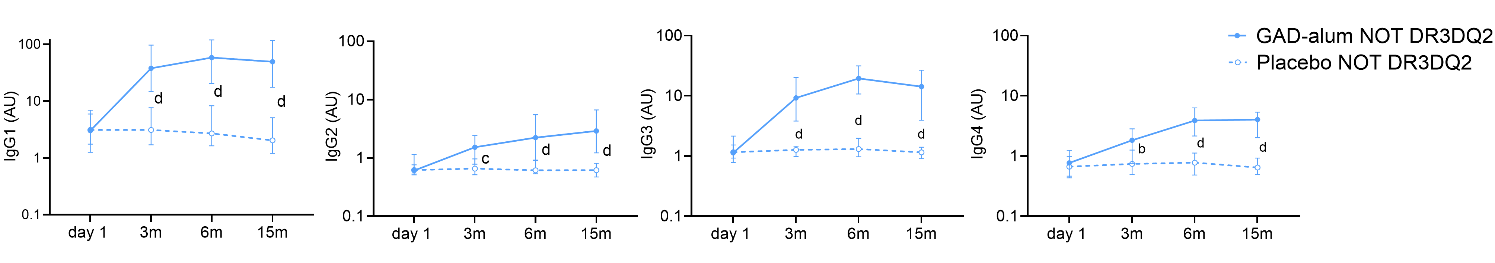

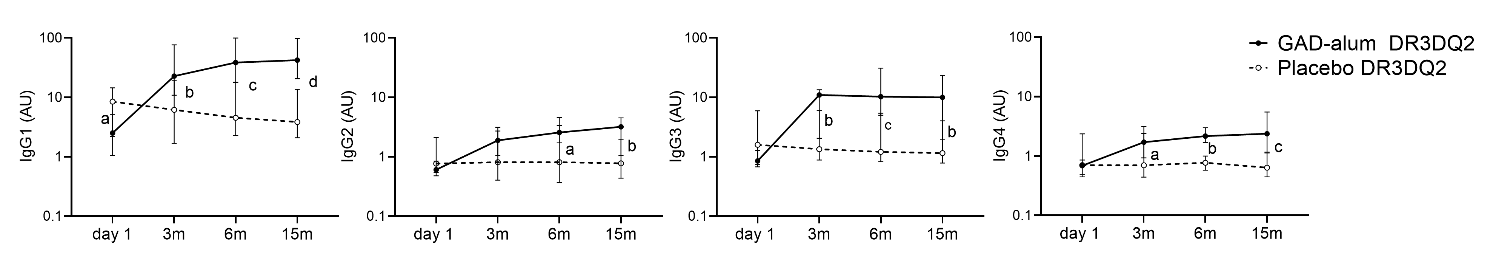
**

**C**

**B**

**Supplementary Figure 2.** Median levels of IgG1, IgG2, IgG3 and IgG4 GADA subclasses from day 1 to 15 months shown as arbitrary units (AUs). **(A)** Median levels of GADA subclasses in GAD-alum (smooth line, n=29) and Placebo (dashed line, n=19) treated patients with DR3DQ2 haplotype. **(B)** Median levels of GADA subclasses in GAD-alum (smooth line, n=27) and Placebo (dashed line, n=32) treated patients without DR3DQ2 haplotype. **(C)** Median levels of GADA subclasses in GAD-alum treated patients with (black, n=29) or without (blue, n=27) DR3DQ2 haplotype. Horizontal lines represent the median and error bars indicate 95% CI. Differences between groups were calculated using Mann-Whitney unpaired test. a, b, c, d: p<0.05, p<0.01, p<0.001, p<0.0001.

**
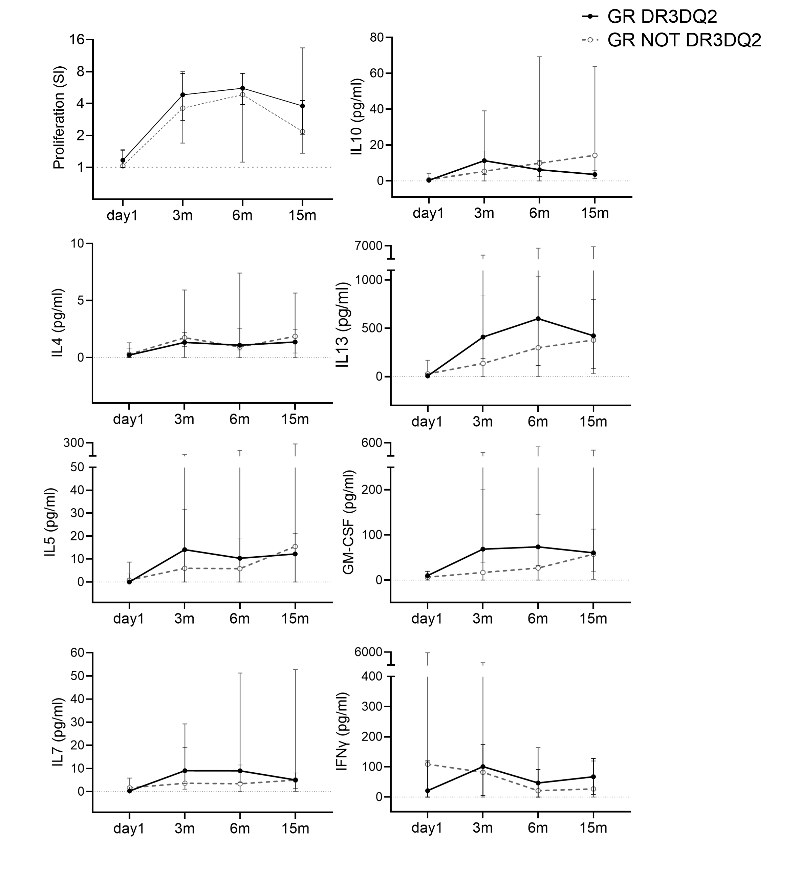

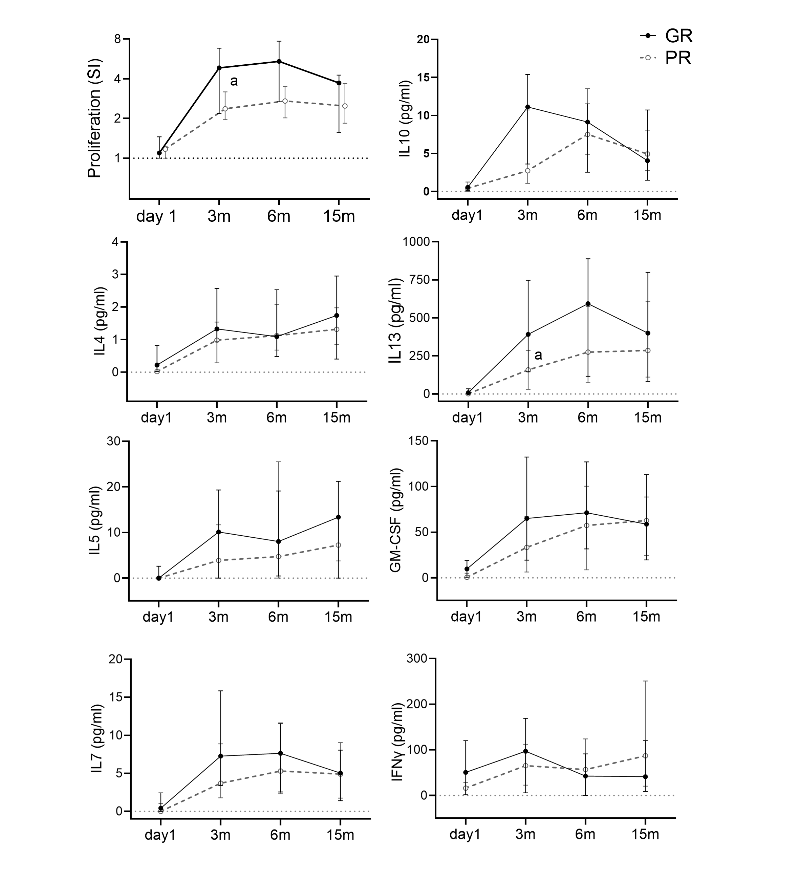
**

**B**

**A**

**Supplementary Figure 3:**GAD_65_-induced cytokine secretion and Proliferation. **(A)** GAD-alum group stratified into Good Responders (GR, n=23, loss < 30% AUC, smooth line) and Poor Responders (PR, n = 32, loss ≥ 30% AUC, dashed line) according to their C-peptide preservation at 15 months. **(B)** Good Responders (GR, n=23, loss < 30% AUC) in the GAD-alum group with (n=17, smooth line) and without (n=6, dashed line) DR3DQ2 haplotype. Proliferation is expressed as stimulation index (SI), calculated from the mean of triplicates in the presence of GAD_65_ divided by the mean of triplicates with medium alone. Cytokine secretion detected by Luminex in PBMCs supernatants after 7-days culture in presence of GAD65 (5 µg/ml). GAD_65_-induced IL4, IL5, IL7, IL10, IL13, IFNγ and GM-CSF are given after the subtraction of the spontaneous secretion from each individual and expressed as pg/ml. Horizontal lines represent the median, and error bars indicate 95% CI. Error bars indicate 95% CI. Differences within the same group were calculated using Wilcoxon paired test and differences between groups were calculated using Mann-Whitney unpaired test. a, b, c, d: p<0.05, p<0.01, p<0.001, p<0.0001.
